# Toward General Design of Mn-Based Layered Oxide Cathodes for Sodium-Ion Batteries: From Thermodynamic Principles to Entropy Engineering

**DOI:** 10.3390/molecules31050836

**Published:** 2026-03-02

**Authors:** Li Dong, Xiang-Yu Qian, Jian Xiong, Yi-Han Zhang, Xing Wang, Jing-Yi Ding, Fa-Jia Zhang, Jia-Qi Shen, Qi-Rui Zhang, Yong-Gang Sun

**Affiliations:** School of Chemistry & Chemical Engineering, Yancheng Institute of Technology, Yancheng 224051, China; dongli@stu.ycit.edu.cn (L.D.); qianxy05@outlook.com (X.-Y.Q.); xiongjian1917@163.com (J.X.); ruarua0505@163.com (Y.-H.Z.); wang92643864@163.com (X.W.); djy11062026@163.com (J.-Y.D.); zhangfj3219@163.com (F.-J.Z.); shenjq@stu.ycit.edu.cn (J.-Q.S.); zqr050216@163.com (Q.-R.Z.)

**Keywords:** sodium-ion battery, Mn-based layered oxides, rational design, machine learning

## Abstract

Mn-based layered oxide cathodes are pivotal for advancing sodium-ion batteries, yet their practical deployment is hindered by structural instability and complex phase transformations during cycling. This review provides a systematic overview of recent strategies aimed at rational design and performance enhancement of these materials. It begins with fundamental thermodynamic principles governing phase formation, particularly P2/O3 structural dichotomy, and highlights the critical roles of sodium content, transition metal chemistry, and ionic potential in determining crystal stability. The emergence of high-entropy engineering is examined as a powerful approach to suppress detrimental phase transitions through configurational entropy stabilization, lattice distortion, and synergistic multi-element interactions. Furthermore, the integration of machine learning with multidimensional descriptors including electronegativity-weighted entropy and cationic potential enables more accurate predictions of phase behavior in complex compositional spaces. The review also highlights the decisive influence of synthesis protocols, where precise control over calcination conditions, atmosphere, and local elemental distribution enables the formation of targeted phase architectures, such as P2/O3 intergrowth, which exhibit superior electrochemical robustness. Collectively, these advances illustrate a shift from empirical trial and error toward a theory-guided, data-informed framework for designing high-performance layered oxide cathodes.

## 1. Introduction

With the accelerated advancement of global energy structure transformation, lithium-ion batteries have become the dominant technology in portable electronic devices and electric vehicles due to their high energy density and long cycle life [1]. However, the limited reserves of lithium resources and their uneven geographical distribution have led to a continuous increase in raw material costs, severely restricting the economic feasibility and sustainable development of large-scale energy storage systems [2,3]. Against this backdrop, sodium-ion batteries, with their abundant reserves of sodium resources, wide geographical distribution, and similar working principles to lithium-ion batteries, are regarded as one of the most promising next-generation energy storage technologies [4,5,6,7].

In the sodium-ion battery system, the cathode material is the core factor determining the battery’s energy density, cycle life, and cost [5,8]. Among the numerous candidate materials, manganese-based layered transition metal oxides have become the leading candidate for commercial applications due to their high theoretical capacity, mature synthesis process, and good voltage matching [9,10]. Based on the stacking sequence of oxygen layers and the coordination environment of Na^+^ ions, these layered oxides are primarily classified into two structural types: O3 and P2 phases [11,12]. The O3 phase features ABCABC oxygen stacking with Na^+^ residing in octahedral sites and typically exhibits a high sodium content (x > 0.7), enabling high specific capacity; however, it suffers from sluggish Na^+^ diffusion kinetics and complex multi-step phase transitions during (de)intercalation. In contrast, the P2 phase adopts ABBA oxygen stacking with Na^+^ in trigonal prismatic sites, providing larger interlayer spacing and faster ion transport. Nevertheless, its lower sodium content (x ≈ 2/3) inherently limits capacity, and it is prone to detrimental phase transformations—such as P2 → O2 or P2 → Z—under high-voltage charging or deep desodiation, resulting in significant volume changes and structural degradation. This fundamental trade-off between high capacity (favored by O3) and superior kinetics/structural resilience (offered by P2) has spurred the rational design of P2/O3 intergrowth architectures that synergistically integrate the merits of both phases. Among these, P2-type manganese-based oxides have attracted particular attention due to their low cost, earth abundance, and environmental compatibility. However, their practical deployment remains hindered by two critical challenges: (i) the Jahn–Teller distortion induced by Mn^3+^ during cycling, which causes severe lattice distortion and eventual structural collapse; and (ii) irreversible phase transitions (e.g., P2 → O2) under high-voltage or deeply desodiated conditions, leading to abrupt volume changes and electrode failure [13,14].

To address these challenges, research efforts have evolved from conventional single/dual-element doping toward a more systematic ‘universal design’ strategy [15,16,17]. This paradigm aims to establish unified physicochemical descriptors to guide the rational design of materials, thereby overcoming the long-standing hurdles associated with phase instability, compositional complexity, and synthetic inaccessibility in layered oxides [18].

In this review, we adopt several commonly used, comprehensive and universally applicable design frameworks that effectively bridge fundamental thermodynamics with experimental realizability in layered oxide cathodes for sodium-ion batteries. We begin by elucidating the crystallographic foundations and thermodynamic principles governing phase formation—particularly the P2/O3 structural landscape—and delineate the key factors dictating phase stability. Building on these fundamentals, we systematically describe the structure–property relationships of transition metal constituents, with particular emphasis on high-entropy engineering as a powerful strategy to stabilize desirable phases and suppress detrimental transitions. Complementing this materials-centric perspective, we integrate data-driven approaches, leveraging machine learning to validate, refine, and extend classical thermodynamic descriptors across complex compositional spaces. Finally, we address the critical role of synthesis—highlighting how precise control over processing parameters, such as calcination conditions, atmosphere, and cation ordering, enables the targeted realization of theoretically designed architectures and their optimal electrochemical performance. Together, these interconnected pillars form a cohesive roadmap for the rational development of next-generation layered oxide cathodes.

## 2. Thermodynamic Foundations and Phase Design Criteria

The electrochemical performance of sodium-ion layered oxides (Na_x_TMO_2_) is closely related to the microscopic configuration of their crystal structure. During the charging and discharging process, the exodus and incorporation of sodium ions are accompanied by changes in the electrostatic interaction between the transition metal layer and the oxygen layer, thereby determining the phase structure (P2 or O3) and stability of the material [19,20]. This section will elaborate from three aspects: the crystal stacking mechanism, thermodynamic criteria, and the special structural effects of manganese-based materials.

### 2.1. Crystal Structure Foundation and Stacking Mechanism

**Figure 1:**
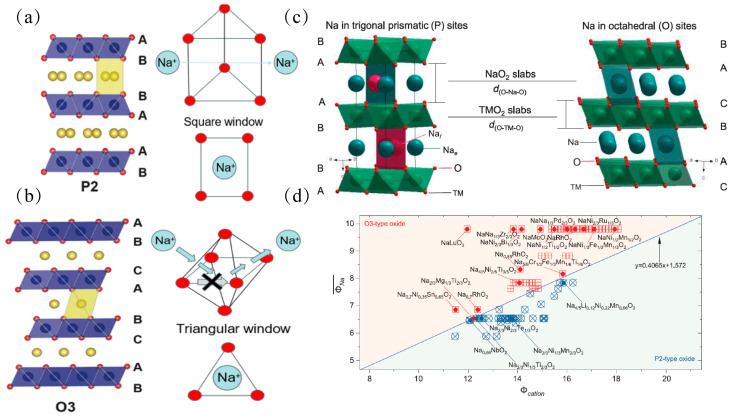
(**a**) Schematic diagram of the P2-type layered oxide structure, along with the path for sodium ion transmission. (**b**) Schematic diagram of the O3-type layered oxide structure, and the path for sodium ion transmission [47]. (**c**) Schematic illustration of crystal representative P2-type (hexagonal) and O3-type (rhombohedral) layered oxides. (**d**) Cationic potential of representative P2- and O3-type Na-ion layered oxides considering the Na content, oxidation state of transition metals, and TM composition [48]. Common criteria and Phase Stability Boundary.

The structure of sodium-ion layered oxides is formed by alternating stacking of [TMO_2_] transition metal layers and sodium ion layers [21,22]. Depending on the coordination environment of sodium ions (octahedral or trigonal prismatic) and the number of the minimum stacking unit, two main structures, O3 and P2 [23], are formed as shown in Figure 1a–c.

The main difference between O3 and P2 lies in the stacking mode. In the O3 phase (ABCA stacking), sodium ions occupy the octahedral voids, and the stacking sequence repeats every three oxygen layers; while in the P2 phase (ABBA stacking), sodium ions occupy the trigonal prismatic voids, and the stacking sequence repeats every two oxygen layers (Figure 1a,b). The main factors influencing the stacking mode are sodium content and the type of transition metal. Sodium content plays a dominant role in influencing the stacking mode as shown in Table 1. When the sodium content (x) is less than 0.7, the P phase structure is preferred, and when the sodium content (x) is greater than 0.7, the O phase structure is preferred. This is because at low sodium contents, the wider sodium layer spacing in the P2 structure can effectively reduce the Coulomb repulsion between sodium ions, exhibiting square-shaped sodium storage vacancies, and from an energy perspective, this structure minimizes the electrostatic repulsion energy of the system, making it thermodynamically more favorable. Conversely, the O3 phase can provide a higher packing density at high sodium concentrations. A large number of sodium ions (Na^+^) brings a dense positive charge. The more compact stacking structure of the O3 phase means that the negatively charged oxygen ion (O^2−^) layers can more effectively approach the sodium ion layers, achieving better charge neutralization and enhancing the lattice energy of the crystal, exhibiting triangle-shaped sodium storage vacancies, making the system more stable [11]. At this time, the supporting strength requirement of the TM-O layer is relatively reduced because the compact stacking structure is inherently more stable. This phase transformation rule induced by sodium content (such as the transition from P2 to O3) is a fundamental physical and chemical parameter that must be considered when designing layered oxides.

### 2.2. Thermodynamic Criteria for Phase Stability

In the design of sodium-ion layered oxides (Na_x_TMO_2_), achieving precise control over the crystal structure is the core to achieving the “universal design” goal. This control not only relies on the adjustment of the stoichiometric ratio but also needs to be based on a solid thermodynamic foundation. By combining Table 1, it can be seen that the sodium ion content is an important descriptor for describing the structure of layered materials, and it is also a more universal descriptor—the ionic potential ($\Phi_{TM}$) can also be used as the main criterion to predict phase stability, and combined with the configurational entropy effect introduced by the high-entropy strategy, to achieve precise control over the composition of the P2/O3 phase.

#### 2.2.1. Common Criteria and Phase Stability Boundary

As shown in Table 1, even if the sodium content is similar, different combinations of transition metals (TM) will result in the final product adopting either the P2 or O3 phase (for example, Na_2/3_Ni_1/3_Mn_2/3_O_2_ [49] is the P2 phase, while NaNi_1/3_Fe_1/3_Mn_1/3_O_2_ [50] is the O3 phase). This indicates that, in addition to the sodium content, the intrinsic physical and chemical properties of the TM ions have a decisive influence on the structural stability. To address this issue, Hai et al. [44] formed high-sodium P-type structures using different transition metal species, and these structures coexist with the O-phase structure. Lin et al. [46] synthesized the material Na_x_Li_0.05_Mn_0.55_Ni_0.3_Cu_0.075_Mg_0.025_O_2_, reducing Jahn–Teller active ions, enhancing the reversibility of phase transformation, and improving the diffusion kinetics, thereby enhancing the cycling and rate capabilities of the P2/O3 nano-growth intercalated cathode. Therefore, finding a unified descriptor to interpret the influence of alkali metal ions on the layered oxide structure is an urgent problem to be solved.

The valence state of transition metals (TM) is a key determinant of the electrostatic interaction between the TM and oxygen layers. High-valence TMs (e.g., Ti^4+^ [51], Zr^4+^ [52], Sn^4+^ [53]) possess strong polarizing power, leading to enhanced covalent bonding with oxygen. This draws electron density away from the oxygen anions, stabilizing the relatively open ABBA-stacked P2 structure. In contrast, low-valence cations (e.g., Li^+^ [54], Cu^2+^ [28]) exhibit weaker interactions with oxygen, resulting in higher electron density on the oxygen sites. This favors the formation of the densely packed ABC-stacked O3 phase.

Building upon the radius ratio between the TM layer and the alkali metal layer in P2/O3 phases, Zhao et al. [55] established a “composition-structure” map (Figure 1d). Generally, a critical radius ratio threshold exists: systems with an average ratio above this threshold are thermodynamically favored to form the P2 phase, while those below tend to adopt the O3 phase. This criterion provides a quantitative guideline for designing Mn-based materials—for instance, by incorporating high-valence dopants such as Ti or Zr to stabilize the P2 phase.

#### 2.2.2. High-Entropy Strategy and Configuration Entropy Stabilization Effect

With the upgrading of material design concepts, the high-entropy strategy (HES) introduces a new thermodynamic dimension for phase stability control—configurational entropy ($S_{config}$).

According to the Gibbs free energy formula (G=H−TS), in a multi-component system, mixing multiple transition metal cations significantly increases configurational entropy. Under high-temperature synthesis conditions, the contribution of the *TS* term increases, thereby reducing the total Gibbs free energy of the system. This “entropy-driven” effect can overcome the enthalpy increase caused by lattice distortion (H), allowing unstable metastable structures (such as a specific biphasic ratio of P2/O3) to exist stably.

It has been found that during electrochemical cycling, high-entropy materials exhibit a unique “lattice hysteresis effect” and “cocktail effect”. High configurational entropy can effectively inhibit the long-range ordered rearrangement of transition metal ions, thereby suppressing harmful phase transitions [56] (such as the slip phase transition from P2 to O2) during charging and discharging. This kinetic hindrance brought about by the entropy effect essentially increases the activation energy barrier of phase transitions, enabling the material to maintain the integrity of the main structure even in a deeply discharged state. Due to the special nature of high-entropy materials, configurational entropy has also become an important descriptor.

#### 2.2.3. Multidimensional Thermodynamic Descriptor

In order to achieve more precise “universal design”, relying solely on cationic potential or simple tolerance factors is no longer sufficient to describe the phase behavior of complex and diverse systems. Therefore, considering multiple descriptors has become an inevitable approach for accurately predicting the structure of layered oxides, especially with the rise of emerging technologies such as machine learning, making the consideration of multiple descriptors an important issue in current development [57].

A single descriptor (such as transition state ion radius) has limitations when applied to complex high-entropy systems [58]. Therefore, it is necessary to combine multiple descriptors—such as those listed in Table 2—to construct a multidimensional feature space and employ advanced techniques like machine learning for collaborative analysis, thereby enabling accurate prediction.

## 3. Elemental Chemistry and Multielement Synergy

The electrochemical performance of sodium-ion layered oxides not only depends on their macroscopic morphology and preparation process [4] but, at a deeper level, is determined by the intrinsic chemical properties of the constituent elements and the synergistic control of multi-scale structures [59]. By precisely designing the composition and ratio of transition metal (TM) elements and introducing novel materials-design concepts such as the high-entropy strategy, it is possible to achieve synergistic optimization of material structural stability and ion-transport kinetics—from the atomic scale to the mesoscopic scale [60,61]. This chapter will focus on the structure–property relationships of transition metal elements and the rational design principles underlying the high-entropy strategy, systematically analyzing how elemental chemistry can guide the rational design of material structures.

### 3.1. Structure–Activity Relationships of Transition Metal Elements

Transition metal elements act as redox-active centers in layered oxides. Their electronic structure [62], ionic radius [63], valence state [64], and bonding characteristics [65] with oxygen directly determine the specific capacity, working voltage [66], structural reversibility [67], and cycling stability of the materials [68]. Establishing the structure–activity relationship of transition metal elements is the foundation for precisely controlling the performance of materials.

**Figure 2:**
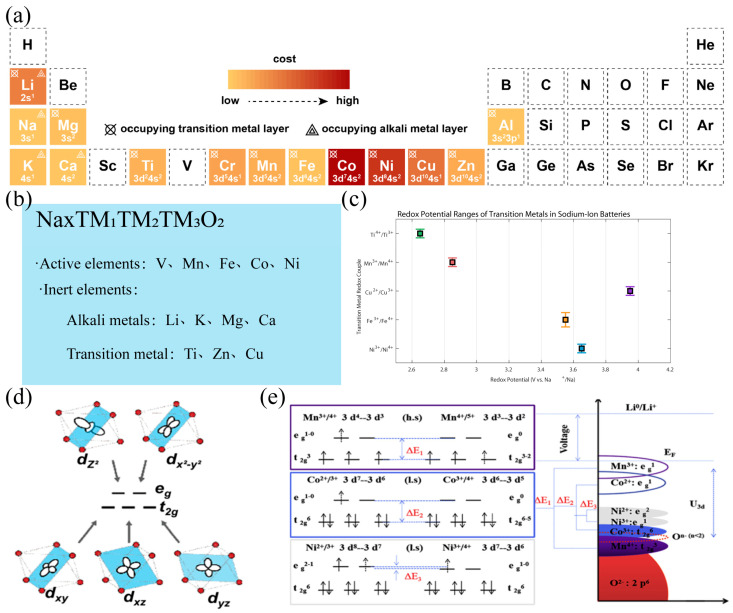
(**a**) The positions of common impurity elements in the periodic table [74]. (**b**) Common dopant elements. (**c**) Common active potentials of doping elements. (**d**) Crystal field splitting of d orbitals in an octahedral environment. Reproduced with permission. (**e**) Electronic state diagram of Mn-Co-Ni 3d orbital ions in the TMO_6_ octahedral structure at different oxidation states [75].

Transition metals can be classified into active transition metals, which participate in redox reactions within the working voltage range (such as Ni^3+^/Ni^4+^ [69], Mn^3+^/Mn^4+^ [70], etc.) and inert transition metals, which do not participate in redox reactions within the working voltage range (such as Ti^4+^ [71], Zn^2+^ [72], Cu^2+^ [73], etc.), as shown in Figure 2a,b.

**Figure 3:**
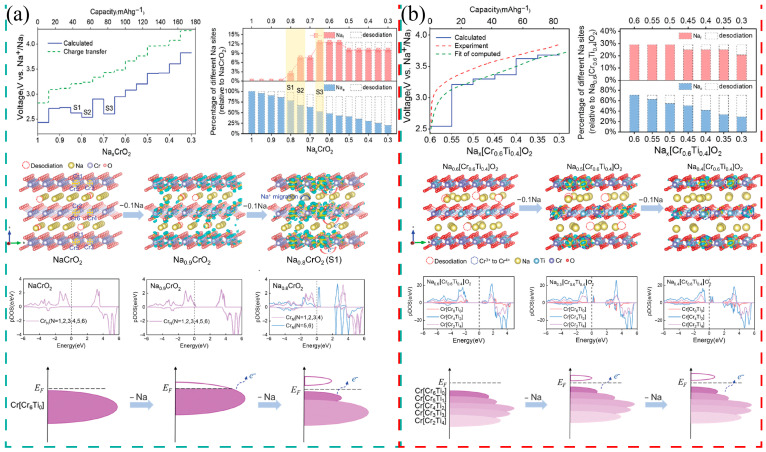
Analysis of calculation resultsof (**a**) P2-NaCrO_2_ [76], (**b**) Na_x_[Cr_0.6_Ti_0.4_]O_2_ (x = 0.6, 0.55, 0.5, 0.45, 0.4, 0.35, 0.3) [77], including calculated voltage and charge transfer behavior, structural evolution with charge density difference, variations in partial density of states, and energy level changes within the structure.

The 3d electron configuration of transition metals determines the number of electrons that can participate in redox reactions and the potential platform, as shown in Figure 2c. The Ni^3+^/Ni^4+^ redox pair usually lies in the higher voltage range (>4.0 V vs. Na^+^/Na), contributing high energy density, while the Mn^3+^/Mn^4+^ pair is mostly distributed in the 3.5–4.0 V range; although the voltage is lower, it has a good structural supporting effect. Although Fe^2+^/Fe^3+^ has a theoretical high capacity, it is prone to irreversible phase transformation during deep deionization. Yang et al. [76] found that P2-NaCrO_2_ would produce obvious multiple voltage platforms, as shown in Figure 3a, while no such phenomenon was observed in P2-Na_0.6_[Cr_0.6_Ti_0.4_]O_2_ (Figure 3b). This is due to the disordered arrangement of transition metals. The research found that, as shown in the process of sodium ion and electron transfer (i.e., during the redox process), the delocalization of electrons and ions is the root cause of the formation of the platform. Different Na sites (a, b, c) successively deplete at each stage, demonstrating spatial selectivity. Along with sodium removal, Cr^3+^ is oxidized to Cr^4+^, the oxygen framework undergoes distortion, and even partial oxidation of O^2−^ may occur, leading to structural instability. The projected density of states (pDOS) and energy band structure further confirm that the 3d orbitals of Cr dominate the charge compensation, and there is strong hybridization between O 2p and Cr 3d. In contrast, Ti doping significantly improves the material performance: the voltage curve is smoother, the sodium removal path is more uniform, and the phase transition is effectively suppressed. Due to the stable 3d^0^ electronic configuration of Ti^4+^, it does not participate in redox reactions and only acts as a “structural stabilizer”, thereby reducing lattice strain and the propensity of oxygen oxidation. pDOS shows that the introduction of Ti does not contribute electronic states near the Fermi level, while Cr still bears the main redox activity.

Mn^3+^ (t_2_g^3^eg^1^) is a typical Jahn–Teller active ion. During charging and discharging, it is prone to cause axial elongation distortion of the octahedron, resulting in local stress accumulation, micro-crack formation, and capacity degradation, as shown in Figure 2d. To suppress this effect, partial substitution with non-active or weak Jahn–Teller active transition metals can be introduced to achieve electron structure homogenization, thereby alleviating local strain.

The degree of orbital hybridization between transition metals and oxygen affects the covalency of the TM-O bond, thereby regulating the electronic conductivity and surface reconstruction behavior of the material. Highly covalent systems (such as those containing strong hybridization of Ni/O or Co/O) usually have better electron transport ability, as shown in Figure 2e, but they may also exacerbate the surface oxygen evolution reaction (OER), leading to a decrease in air stability. Therefore, while pursuing high conductivity, surface passivation or the introduction of high electronegative elements (such as F doping) should be used to enhance interface stability to meet the requirements of aqueous processing and long-term storage [78]. The traditional “tolerance factor” model [79] originated from the perovskite structure, with its core assumption being the geometric matching of ionic radii. However, the structural stability of layered oxides is not solely determined by atomic sizes. In complex multi-component systems, differences in electronic structures and the contribution of mixed entropy pose new requirements for the adaptability of the lattice, making the traditional model based on geometric size no longer applicable.

Therefore, effective descriptors need to go beyond simple geometric considerations and establish new descriptors that can reflect electronic interactions and the configurational disorder degree. Utilizing the core idea of tolerance factors, an attempt was made to distinguish P2 and O3 types of structures based on the ratio of interlayer distances between alkali metals (d(O-Na-O)) and transition metals (d(O-M-O)). Zhao et al. [55] summarized that a ratio of approximately 1.62 can be used as an indicator to distinguish P2 and O3 structures, as shown in Figure 1d. Thus, a clear explanation can be provided for the stabilizing effect of large-radius ions (such as Sn^4+^ and Sb^5+^). It can effectively widen the TM interlayer spacing and is more inclined to form a P-type structure. However, overly large ions may disrupt local coordination symmetry and cause local distortions. Therefore, the collaborative optimization of ionic charges and radii is an effective way to achieve structural stability and unified dynamic performance.

### 3.2. Multielement Synergy to Suppress Unfavorable Phase Transformation

The single-component doping strategy plays a crucial role in stabilizing the layered structure. However, due to its limited component quantity and other characteristics, the effect is relatively narrow and cannot meet the goal of simultaneously optimizing multiple properties. Therefore, the high-entropy strategy has been widely valued. That is, by introducing five or more main elements and mixing them in nearly equal molar ratios to form a single-phase solid solution structure, the “lattice distortion effect”, “slow diffusion effect”, “cocktail effect”, and “high configurational entropy stabilization effect” are utilized to achieve coordinated control of the multi-scale structure of the material. Currently, the high-entropy doping strategy has become an important paradigm for the design of the next-generation layered oxides.

**Figure 4:**
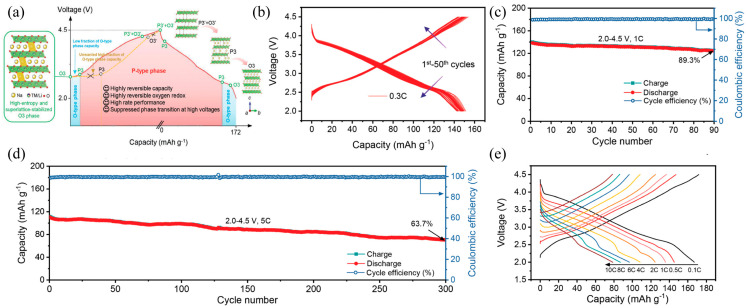
(**a**) Schematic illustration of charge/discharge behaviors for high-entropy and superlattice-stabilized O3-type cathodes proposed in this work, where the O3–P3 phase transition at the low-voltage region is facilitated and the P3–O3 phase transition at the high-voltage region is suppressed. Electrochemical performance of NaLFCNM cathode. (**b**) The charge–discharge profiles at a 0.3C rate for 50 cycles. (**c**,**d**) Long-term cycling performance at (**c**) 1C rate and (**d**) at 5C rate. (**e**) Charge–discharge curves at different cycling rates [80].

In the high-entropy system, multiple transition metal ions are randomly distributed in the lattice, creating a “chemical disorder” environment. This disordered structure significantly increases the energy barrier for atomic migration, thereby inhibiting the long-range ordered rearrangement of the transition metal layers during sodium desorption. Yao et al. [80] employed the stabilizing effect of high-entropy superlattices to synthesize the cathode material Na_2/3_Li_1/6_Fe_1/6_Co_1/6_Ni_1/6_Mn_1/3_O_2_. Their work demonstrated that this material exhibits not only excellent electrochemical performance, but also high phase stability and suppressed oxygen reduction (Figure 4a). As shown in Figure 4b–e, the material delivers a first-cycle capacity of 152 mAh·g^−1^ at 0.3C and 171.2 mAh·g^−1^ at 0.1C. After 90 cycles at 1C, a capacity retention of 89.3% is maintained, indicating remarkable cycling stability. Furthermore, Figure 4e reveals that a capacity of 80 mAh·g^−1^ is retained even at 10C, suggesting promising potential for high-rate operation. The lattice retardation effect also contributes to enhanced mechanical robustness. Liu et al. [81] leveraged the disordered structure and multi-ion cocktail effect of high-entropy materials to develop a cobalt-free cathode, Na_0.9_Ni_0.3_Fe_0.2_Mn_0.3_Ti_0.1_Cu_0.05_Sn_0.05_O_2_, with deep discharge capability. By extending the cut-off voltage to 4.2 V, they achieved a high energy density. It was further shown that in the deeply charged state, slab sliding inhibits the P3 to OP2 phase transition and confers resistance to humid air. The material exhibits quasi-zero strain characteristics with a minimal volume change of 1.29%, good rate performance (101.3 mAh·g^−1^ at 10C), and long-term cycling stability (90.7% capacity retention after 400 cycles at 5C). In a full-cell configuration, it achieves a high energy density of 299.3 Wh·kg^−1^ with only 3% capacity decay after 200 cycles at 1C.

The lattice retardation effect is a unique dynamic property of high-entropy materials. Due to the different sizes and bonding strengths of various ions, strain traps are formed in local areas, hindering dislocation slip and grain boundary migration. This effect enables the material to maintain particle integrity during repeated volume expansion/contraction processes, significantly reducing the initiation and propagation of microcracks.

### 3.3. Cocktail Effect for Multifunctional Integration

The cocktail effect refers to the synergistic action of multiple elements resulting in new functions that exceed the performance of a single component. In high-entropy design, the following functional integration can be achieved: (1) Structural support: large-radius ions (such as Sn^4+^ [62], Ti^4+^ [82]) act as lattice pillars to maintain interlayer spacing; (2) electronic conductivity enhancement: Ni/Co contribute high electron density [83]; (3) Jahn–Teller inhibition [83]; and (4) surface stabilization: surface segregated elements (such as Mg^2+^, Al^3+^) form a stable CEI layer [84].

**Figure 5:**
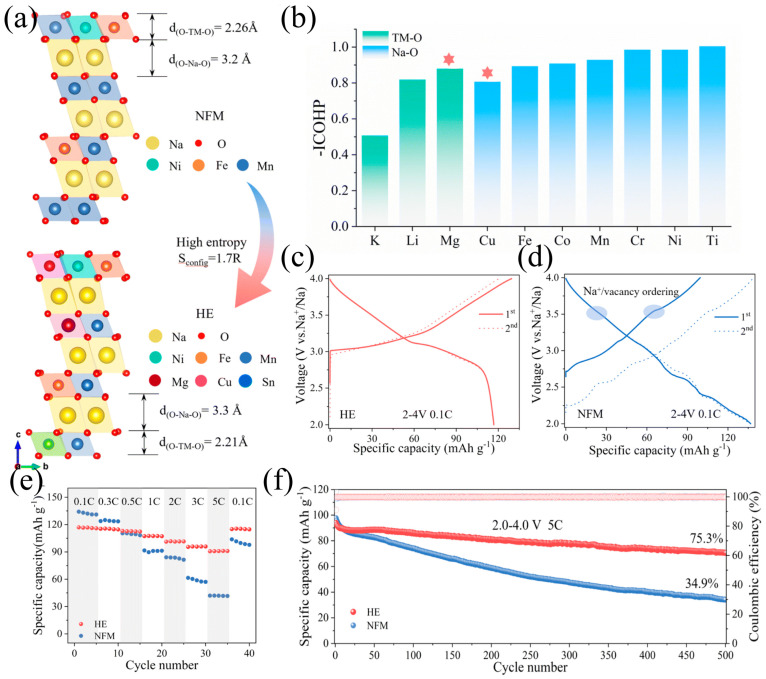
(**a**) Structural comparison of NFM and HE materials. (**b**) Variation in bond strengths (TM–O and Na–O) in the Na–O–TM configuration after alkali metal and 3d transition metal substitution at the Mn site. Comparison of electrochemical performance of electrode materials. (**c**) First charge–discharge curves of HE at a current density of 0.1C within the voltage range of 2–4 V. (**d**) First charge–discharge curves of NFM materials under the same conditions (**e**) Comparison of rate performance of the materials. (**f**) Cyclic performance of HE and NFM at 5C [86].

This “one material with multiple functions” design paradigm provides a new path for achieving universal cathode materials. Ding et al. [85] rationally utilized the cocktail effect in high-entropy sodium-ion batteries to design the cathode material HEO424 (NaNi_0.25_Mg_0.05_Cu_0.1_Fe_0.2_Mn_0.2_Ti_0.1_Sn_0.1_O_2_), and through experiments, it was proved that by introducing multi-component transition metals (such as Sn^4+^, Ti^4+^, etc.), the interlayer spacing of the high-entropy material HEO424 (TMO_2_) significantly expanded (a = 3.0193 Å, c = 16.065 Å), which had a more stable framework structure compared to the baseline material NFM424 (a = 2.9661 Å, c = 15.989 Å). The expanded Na^+^ transport channels effectively inhibited Jahn–Teller distortion and Na^+^/vacancy ordering and delayed the O3 → P3 phase transition. From the electrochemical performance perspective, the uniform distribution of multiple elements made the charge distribution more uniform and weakened the Na^+^/vacancy ordering. The Na^+^ diffusion coefficient of HEO424 reached twice that of NFM424 and still maintained high diffusion ability after cycling. The ellipsoidal particle morphology provided a thicker Na^+^ transport layer (about 3 μm), significantly improving the rate performance (5C capacity retention rate 82.5%). Moreover, after 200 cycles, the HEO424 particles had no cracks, and the surface only had slight bending of the TMO_2_ layer, while NFM424 showed rock salt phase reconstruction and TM dissolution. The high-entropy design enhanced the TM-O bond energy to inhibit the accumulation of phase transformation stress, ensuring long-term structural stability. Li at el. [86] developed a high-entropy layered oxide system, designated as O3–NaNi_0.2_Fe_0.2_Mn_0.3_Mg_0.1_Cu_0.1_Sn_0.1_O_2_ (HE), through rational compositional design to modulate the cationic radius mismatch and optimize the structural tolerance factor. This strategic engineering of the local coordination environment enhances lattice structural integrity, effectively suppressing detrimental phase transitions—particularly around 3.5 V—during repeated Na^+^ (de)intercalation, thereby significantly improving long-term cycling stability, as evidenced in Figure 5a–d. Furthermore, leveraging the synergistic “cocktail effect” arising from the multi-principal-element configuration, the material exhibits accelerated Na^+^ transport kinetics, attributed to modified electronic structure and reduced diffusion energy barriers. This kinetic enhancement enables the HE cathode to deliver high-rate capability while concurrently maintaining a high energy density and excellent capacity retention over extended cycling, even under high-current operation, as demonstrated in Figure 5e,f.

### 3.4. Elemental Doping for Modulating Lattice Oxygen Redox Activity

Rational design of cathode materials is typically grounded in thermodynamic principles to stabilize desired crystal structures. However, under high-voltage operation, lattice oxygen is frequently activated to participate in anionic redox processes—commonly referred to as oxygen redox—which substantially boosts energy density but concurrently risks irreversible oxygen loss. Such oxygen release drives structural deviation from the as-designed framework, compromises thermodynamic stability, and ultimately manifests as rapid capacity decay or even collapse of the layered architecture. Consequently, strategic manipulation of lattice oxygen behavior—particularly through elemental doping—has emerged as a pivotal approach to suppress oxygen evolution while promoting reversible oxygen redox activity [87].

High-valence, low-electronegativity cations (e.g., La^3+^ [88], Si^4+^ [89]) exhibit strong electrostatic and covalent coupling with O^2−^ due to their high charge density. This interaction lowers the O 2p band center relative to the Fermi level and increases the formation energy of oxygen vacancies, thereby effectively mitigating irreversible oxygen release and simultaneously enabling stable operation at elevated voltages [88]. Conversely, “oxygen redox activators” such as Li^+^ [90] and Mg^2+^ [91]—owing to their low electronegativity—can locally enrich electron density on adjacent oxygen sites. This facilitates reversible O^2−^/O_n_^−^ (n < 2) redox without triggering molecular O_2_ evolution, thereby unlocking additional capacity while maintaining structural integrity [90].

Beyond direct oxygen stabilization, doping also exerts indirect control by suppressing transition metal (TM) migration—a critical degradation pathway. In high-entropy or multi-element-doped systems, the so-called “lattice pinning effect” impedes TM diffusion into the alkali layer. Since migrated TM ions act as electron traps that destabilize oxidized oxygen species, inhibiting this migration indirectly enhances oxygen redox reversibility. Moreover, dopants with tailored ionic radii or electronic configurations can fine-tune local TM–O–M bond angles and lengths, thereby modulating the oxygen redox potential and steering the reaction pathway toward reversible electron transfer rather than O–O dimerization and O_2_ release [92]. Critically, an optimal doping strategy must strike a delicate balance: reinforcing TM–O bonding to suppress oxygen vacancy formation while retaining sufficient sodium vacancies to ensure fast Na^+^ transport.

## 4. Toward Rational Design: A Paradigm Shift in Materials Optimization

Traditional materials design, which relies on single descriptors such as transition metal valence, often fails to capture the complexity of multi-component systems, rendering trial-and-error approaches prohibitively expensive. This limitation becomes particularly acute with the emergence of high-entropy materials.

To address these challenges, the field is undergoing a paradigm shift from empirical trial-and-error to a rational design methodology encompassing theoretical guidance, computational prediction, and experimental validation. By constructing a multidimensional feature space—integrated with diverse physicochemical parameters—and leveraging advanced data-driven analytics such as machine learning, it is now feasible to accurately predict material properties at the atomic scale.

This chapter systematically outlines structural design based on electronic potential, high-precision computational simulations, and machine learning-driven high-throughput strategies. It establishes an integrated theory-computation-synthesis framework for the physical design of materials, providing a robust methodological foundation for the development of next-generation high-performance cathode materials for sodium-ion batteries.

### 4.1. Structural Design Based on Electronic Potential

**Figure 6:**
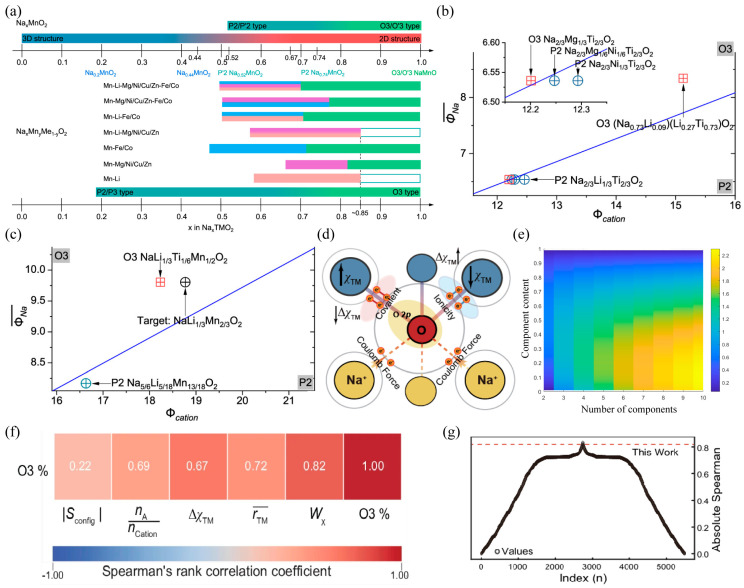
(**a**) Structure composition of Na_x_MnO_2_ and Na_x_Mn_y_Me_1−y_O_2_ compounds [93]. (**b**) Analysis of cationic potentials on P2- and O3-type oxides. (**c**) Designing O3- and P2-type oxides based on the proposed cationic potential [48]. (**d**) Relationship between cationic electronegativity and anionic electron cloud. (**e**) Graph showing the functional relationship between different group numbers, contents and material entropy values (**f**) Spearman’s rank correlation coefficient heat map of materials with different configurational entropy. (**g**) Distribution of Absolute Spearman Values [94].

Electrostatic potential (ESP) is a fundamental physical quantity that describes the distribution of charges within a crystal and the interactions between ions. It directly determines the behavior of sodium ion insertion and removal, the stability of the structure, and the path of phase transformation. By regulating the cooperative relationship between the cation potential and the anion, precise control can be achieved over the stacking mode of the layered structure, the sodium layer spacing, and the migration barrier (Figure 6a) [48].

In the structural design of layered oxide materials for sodium-ion batteries, the cationic potential (Cationic Potential, $\Phi_{cation}$) has been established as the key descriptor determining the crystal stacking structure [48]. This theory integrates the charge and radius information of transition metals (TM) and sodium ions to quantify the electrostatic interactions between layers: the greater the cationic potential, the stronger the electron cloud repulsion between TMO_2_ layers, and the more inclined they are to form the P2-type structure; conversely, higher sodium content or lower cationic potential are conducive to the formation of the O3-type structure. Based on this, researchers constructed a phase diagram of cationic potential and sodium content, as in Figure 3a, achieving a transition from the “trial-and-error method” to “rational design”. For example, to synthesize the target lithium-rich O3 phase material NaLi_1/3_Mn_2/3_O_2_, the design strategy introduced low-ion-potential Ti^4+^ to replace part of Mn^4+^, successfully reducing $\Phi_{cation}$ and stabilizing the O3 structure, as in Figure 6b,c, while in designing high-sodium-content P2 phase materials, the cationic potential was increased by regulating the Mn content, successfully preparing Na_0.75_Li_0.25_Mn_0.75_O_2_ [48]. This rational design paradigm, based on electronic potential and electrostatic environment, not only reveals the quantitative relationship between electronic structure and macroscopic properties but also provides precise theoretical guidance for the development of high-performance sodium-ion battery positive electrode materials.

The traditional design strategy of cationic potential has obvious limitations when dealing with complex multi-component systems. The core drawback lies in its inability to provide reasonable structural prediction guidance for “entropy-dominated” high-entropy materials. Specifically, the cationic potential theory is employed to describe how sodium content influences structural stacking. This is achieved by modulating the oxidation state and ionic radius of transition metals, thereby regulating the electrostatic interactions between layers. However, in high-entropy oxide systems, the formation of phases is not only dependent on the cationic potential but is also governed by complex component and structure relationships. Due to the highly disordered transition metal layers and significant configurational entropy effect in high-entropy systems, the traditional cationic potential method is unable to capture these entropy-driven phase structural features, thus being ineffective in guiding the rational design of high-entropy layered oxides.

### 4.2. Prediction Model for High-Entropy Materials

To break through the limitations of traditional design of sodium-ion layered oxides, Lu et al. [94] proposed a novel rational design strategy based on “electronegativity entropy weight” ($W_{\chi }$) as shown in Figure 6d, and successfully prepared an O3-type high-entropy cathode material with superior electrochemical properties at a low sodium content. In response to the failure of the traditional “cation potential” theory in complex multi-component high-entropy systems, this team innovatively combined electronegativity with configurational entropy and established a quantitative descriptor that can accurately predict the stacking structure (P2/O3) of the material.

$$W_{\chi }=\prod \chi_{Cation}\times \frac{\left|S_{config}\right|}{R}\times \frac{r_{TM}}{r_{A}}$$

Among them $\prod \chi_{Cation}$ represents the cation electronegativity index, which indicates the degree of covalent/ionic nature of the chemical bond and the degree of localization of the electron cloud. It satisfies

$$\prod \chi_{Cation}=\frac{nA}{ncation}\times \chi_{A}\times {∆\chi }_{TM}$$

$\chi_{A}$ represents the average electronegativity difference between oxygen and alkali metals in the AO_2_ structure.

$$\Delta \chi_{TM}=\chi_{0}-\overset{¯}{\chi_{TM}}$$

$\Delta \chi_{TM}$ represents the difference in electronegativity between oxygen and the average value of transition metal ions. $\Delta \chi_{TM}$ is used to quantify the degree of ionic bond formation between transition metal ions and oxygen, defined as the bond polarity index. $\left|S_{config}\right|$ represents the absolute value of configurational entropy. $\frac{r_{TM}}{r_{A}}$ indicates the ratio of ionic radii between the transition metal element and the alkali metal element, representing the geometric spatial hindrance and electrostatic shielding effect.

As shown in Figure 6f,g, by calculating the Spearman rank correlation coefficients between different descriptors and the O3 content (O3%), the system evaluated the predictive ability of each descriptor for the stability of the O3 phase. The results indicated that the correlation coefficient between $|S_{\mathrm{config}}|$ and O3% was 0.22, showing a relatively weak predictive ability, while the correlation coefficient of the electronegativity entropy weight, $W_{\chi }$, was as high as 0.82, being the highest among all descriptors, significantly superior to other parameters. This indicates that $W_{\chi }$ can more accurately reflect the regulatory effect of the distribution of cation electronegativity in the material on the stability of the O3 phase, having excellent physical interpretability and predictive validity. Therefore, it can be regarded as an efficient and reliable descriptor for guiding the rational design of layered oxides with high O3 phase content.

This is actually a comprehensive criterion that measures the balance between “in-layer bonding” and “inter-layer repulsion” in the layered structure. It breaks the traditional empirical rule that solely relies on sodium content to predict the structure, providing a theoretical basis for designing new high-entropy battery materials. Based on this theoretical guidance, by introducing various transition metals (Ni, Cu, Co, Fe, Mn, Ti) to construct a high-entropy environment, the traditional rule that “low sodium content is difficult to form the O3 phase” is broken. The target material with the chemical formula Na_0.67_Ni_0.18_Cu_0.18_Fe_0.18_Co_0.17_Mn_0.17_Ti_0.17_O_2_ was successfully synthesized. Experimental results show that this material not only exhibits excellent air stability (performance does not deteriorate when exposed to air) and thermal stability, but also has excellent electrochemical properties, including a high-capacity retention rate (reaching 93.02% after 200 cycles) and application potential in potassium-ion batteries. This method, through the mode of “theoretical innovation + material verification”, provides an important scientific basis and design paradigm for developing high-stability, low-cost next-generation cathode materials for alkali metal-ion batteries.

### 4.3. The Biphasic Synergy Model as a Method for Designing Highly Stable Cathode Materials

High-entropy oxide cathodes have been widely used as a new type of material, but structural coexistence, as another possibility of high-entropy (structural entropy) materials, has been overlooked. Lee et al. [95] formed a P2/O3 interpenetrating structure by doping Li into the alkali metal layer of NaNi_0.5_Mn_0.5_O_2_, and then prepared Na_1−x_Li_x_Ni_0.5_Mn_0.5_O_2_ cathodes with high-rate performance.

Subsequently, Xu et al. [96] conducted a comprehensive study on the phase structure, interface micro-strain, and electrochemical performance interaction mechanism of the layered structure Na_x_Ni_1/3_Co_1/3_Mn_1/3_O_2_ cathode material. Moreover, through operando high-energy X-ray diffraction, they discovered that the coexisting P2/O1/O3 cathode can inhibit the irreversible P2-O2 phase transformation and simultaneously enhance the structural stability of the O3 and O1 phases during the cycling process.

Further research has revealed that the coexistence of P2 and O3 is not solely dependent on the doping of lithium ions. During this process, various scholars explored the synthesis conditions, making specific changes, as shown in Table 3.

From this, it can be seen that the preparation method of the material, the temperature of the calcination, the doping elements, and the setting of the calcination procedure all affect the structure of the material. Therefore, it is also very necessary to explore the conditions for material synthesis.

### 4.4. Defect Engineering

Defect engineering has emerged as a pivotal strategy for advancing the electrochemical performance of sodium-ion battery cathode materials. While rational structural design provides the foundation for “zero-to-one” breakthroughs, further optimization—such as enhancing Na^+^ diffusion kinetics, reinforcing structural integrity, improving electronic conductivity, and creating additional Na^+^ storage sites—necessitates moving beyond the traditional paradigm of perfect crystallinity [113]. Instead, controlled introduction of lattice defects offers a powerful means to reconfigure material properties at the atomic scale, a strategy increasingly validated as both rational and highly effective [114].

At its core, defect engineering represents a paradigm shift from macroscopic, global thermodynamics to microscopic, local thermodynamic control. Conventional materials design often treats properties as ensemble averages over an ideal lattice governed by bulk thermodynamics. In contrast, defect engineering recognizes that macroscopic behavior is frequently dictated by localized atomic environments and electronic structures modulated by point defects (e.g., vacancies, aliovalent dopants) or extended defects (e.g., dislocations) [113]. Although this perspective resonates with kinetic frameworks that emphasize local configuration evolution, it remains fundamentally constrained by local thermodynamic stability rather than purely kinetic pathways. Consequently, a quantitative understanding of defect-scale thermodynamics is essential for the rational implementation of defect engineering.

From a design standpoint, while defect formation and evolution exhibit pronounced local character, they are ultimately governed by the system’s global thermodynamic landscape. First-principles calculations enable predictive assessment of key parameters—including defect formation energies, ion migration barriers, and local density of states—thereby facilitating the pre-experimental optimization of defect type, concentration, and spatial distribution. A canonical example is the layered oxide cathode Na_x_MO_2_ (x < 1), which inherently embodies a defect-engineered architecture through stoichiometric tuning. This material can be conceptualized as a solid solution of NaTM^3+^O_2_ and TM^4+^O_2_, where the incorporation of tetravalent transition metals (TM^4+^) induces intrinsic Na^+^ vacancies in the alkali layer.

These engineered vacancies confer multiple synergistic benefits: (i) they serve as low-energy diffusion channels that markedly enhance Na^+^ mobility [115]; (ii) TM^4+^ strengthens the covalent bonding between the transition metal and oxygen layers, suppressing interlayer gliding and deleterious phase transitions [116,117] (e.g., P2–O2 or P2–Z) during deep desodiation; and (iii) strategic modulation of the TM^4+^ electronic structure optimizes the band alignment, boosting electronic conductivity while mitigating Jahn–Teller distortions associated with Mn^3+^, thereby enhancing structural resilience [118,119].

However, the impact of defects is non-monotonic and critically dependent on their identity, concentration, and spatial arrangement. For instance, moderate oxygen vacancies or high-valence doping can improve charge transport, but excessive oxygen deficiency destabilizes the oxygen framework, inducing local stress accumulation and accelerating structural collapse [120]. Moreover, defect-rich surfaces often exhibit elevated surface energy, promoting parasitic reactions with the electrolyte that yield a thick, inhomogeneous cathode–electrolyte interphase (CEI), increased interfacial impedance, and potential irreversible phase transformations. Critically, metastable defect configurations may also act as nucleation centers for kinetically favored but electrochemically detrimental phases, leading to rapid capacity fading [121].

## 5. Design of Layered Oxides Based on Machine Learning

**Figure 7:**
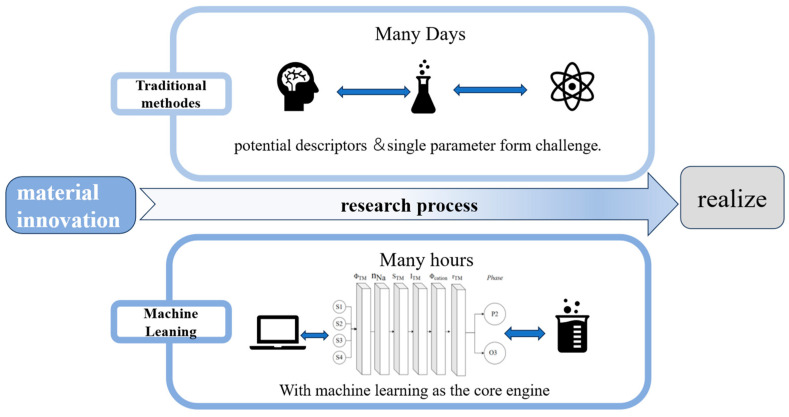
Comparison of the development process of material innovation achieved through traditional approaches and machine learning methods.

As previously discussed, the vast number of potential descriptors, coupled with the combinatorial complexity of even a single parameter, presents a formidable challenge. It is evident that traditional computational methods are impractical for rapidly screening the enormous chemical space (exceeding $10^{5}$ potential combinations). Machine learning (ML), empowered by its superior capability in nonlinear fitting and generalization, has emerged as the core engine for high-throughput materials discovery. It enables a paradigm shift from computation-driven screening to intelligence-driven design, as shown in Figure 7. The application of this work in aqueous electrolyte symmetric supercapacitors has been fully demonstrated. Manickam Minakshi et al. [122] trained the model based on reported relevant data, using ML algorithms to reveal the interrelationships between biomass-derived carbon properties, synthesis conditions, and specific capacitance. This provides strong evidence for the rationality and predictability of the work.

### 5.1. Machine Learning for Rational Exploration of High-Dimensional Compositional Space

**Figure 8:**
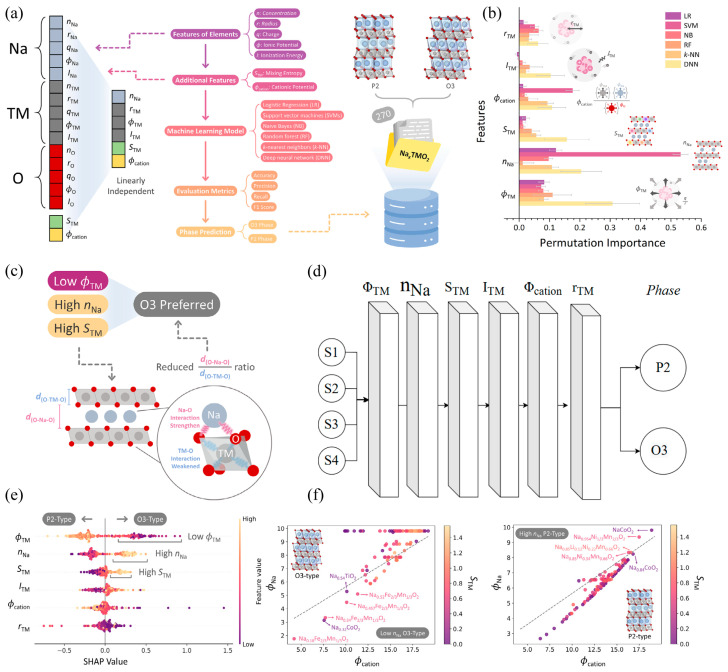
(**a**) Schematic illustration of the workflow in this study, encompassing feature selection, inputting these features into ML models, and predicting the preferred phase of layered oxides. (**b**) Permutation importance of the six features: ionic potential of TM ($\phi_{TM}$), Na concentration ($n_{Na}$), TM mixing entropy ($S_{TM}$) cationic potential ($\phi_{cation}$), TM ionization energy (${Ι}_{TM}$), and TM radius ($r_{TM}$), in each ML-based binary classifier (LR, SVM, NB, RF, k-NN, and DNN). The features are plotted in ascending order of their importance in the DNN model. (**c**) Schematic illustration of the three major criteria favoring the O3 phase and the corresponding changes in TM–O/Na–O interaction strength. (**d**) Schematic diagram of machine learning computational model (**e**) SHAP summary plot for preferred phase prediction in the DNN model, illustrating the SHAP values for each of the six features. (**f**) Distribution of P2-type compositions in the $\phi_{Na}$-$\phi_{cation}$ space, colored by mixing entropy, and distribution of O3-type compositions in the $\phi_{Na}$-$\phi_{cation}$ space, colored by mixing entropy [123].

In order to achieve precise prediction of the phase structure of sodium-ion layered oxides (P2/O3), Wu et al. [123] systematically constructed a set of descriptors with clear physical meanings and mutually independent properties, as shown in Figure 8a. The design of these descriptors follows two core principles, “linear independence” and “physical interpretability”, aiming to avoid information redundancy and enhance the generalization ability and interpretability of the machine learning model. The input vector constructed includes the following multi-scale features, as shown in Table 2.

Subsequently, the machine learning predictions were validated through a multi-scale approach combining electrostatic analysis, density functional theory (DFT), Monte Carlo simulations, and thermodynamic modeling, enabling a deep understanding of the interplay between structural features and phase stability in sodium-ion battery layered oxides. As illustrated in Figure 8b, permutation importance analysis across multiple machine learning models—ranging from linear regression (LR) to deep neural networks (DNN)—reveals that the TM ionic potential ($\phi_{TM}$) is consistently ranked as the most influential feature, with DNN assigning it a relative importance exceeding 0.3. This is followed by Na concentration ($n_{Na}$) and TM mixing entropy ($S_{TM}$), both of which exhibit high predictive significance across models. Notably, while cationic potential ($\phi_{cation}$) and transition metal–transition metal distance ($r_{TM}$) also contribute, their impact is model-dependent and relatively weaker. These results confirm that the electronic structure of the transition metal layer, particularly its ionic potential, plays a dominant role in governing phase selection.

Hence, this further demonstrates that deep neural networks can predict phase stability with exceptionally high accuracy, identifying $\phi_{TM}$, $n_{Na}$, and $S_{TM}$ as the three key descriptors determining phase classification, as shown in Figure 8c,d. Specifically, a lower TM ionic potential, higher Na content, and higher mixing entropy are strongly correlated with the formation of the O3 phase. In contrast, higher $\phi_{TM}$ favors the P2 phase due to stronger electrostatic repulsion between Na^+^ and TM^4+/3+^ ions, which destabilizes the close-packed O3 stacking. Moreover, Na–TM and Na–Na interactions emerge as the primary drivers of phase stability, both of which are highly sensitive to Na concentration. Crucially, $\phi_{TM}$ was determined to be the decisive factor modulating the Na–TM interaction, thereby directly influencing the energy landscape of the system. This is evident in Figure 8e,f, where lower $\phi_{TM}$ leads to reduced Coulombic repulsion between Na^+^ and TM, stabilizing the O3 structure, while higher $\phi_{TM}$ promotes the P2 phase via enhanced charge ordering and lattice strain.

### 5.2. High-Throughput Screening and Discovery of New Materials

At present, machine learning has become an important tool for predicting and screening new materials. Nishant Mishra et al. [124] adopted a dual transition metal doping strategy and used a machine learning model (XGBoost) to predict the average voltage of 650 candidate compounds. The model training used the physical and electronic characteristic parameters of elements, and the test achieved an R^2^ of 0.85 and an RMSE of 0.41, showing good performance. Finally, seven new quaternary and quinary compounds were selected, with their predicted AV ranging from 2.8 to 4.4 V, and the error verified by DFT was within 3–15%. In addition, the phase stability, electronic structure, magnetism, dynamic stability, and diffusion activation energy of these materials were analyzed through DFT and AIMD (Ab Initio Molecular Dynamics) techniques.

**Figure 9:**
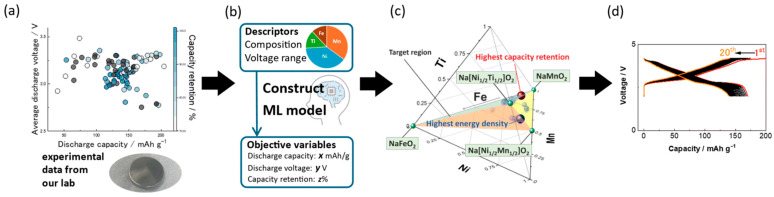
The process of exploring new NaTMO_2_ cathode materials for sodium-ion batteries using ML. (**a**) Experimental dataset utilized in this study, (**b**) construct machine learning model, (**c**) explore promising compositions, (**d**) experimentally verify the electrochemical properties of the resulting compositions [125].

Saaya Sekine et al. [125] pioneered a data-driven approach that integrates experimental data with machine learning (ML) to accelerate the design of sodium-ion cathode materials. By training a predictive model on a curated dataset, they accurately estimated the gravimetric energy density of Na[Mn_0.36_Ni_0.44_Ti_0.15_Fe_0.05_]O_2_, achieving close agreement between predictions and experimental validation, thus demonstrating the practical utility of ML in guiding high-performance battery development. Their systematic methodology, illustrated in Figure 9a–d, encompasses data acquisition from both new experiments and literature, feature engineering to derive physically meaningful descriptors, rigorous data curation to ensure consistency, supervised model training to map compositional features to target properties, and final prediction with objective validation. This iterative and scalable workflow highlights the generalizability of the approach. With the rapid advancement of deep learning and foundation models capable of multimodal reasoning, ML integration is evolving from a supportive tool into a central paradigm for inverse design in materials science, promising to significantly shorten discovery cycles, reduce trial-and-error experimentation, and enable the rational design of complex multicomponent systems beyond the reach of conventional methods.

### 5.3. The Development of Machine Learning for Sodium-Ion Batteries

In the current era of rapid development of machine learning algorithms, traditional machine learning methods (such as decision trees [126], neural networks [123]) still highly rely on efficient feature descriptors to achieve accelerated computation and accurate prediction. Therefore, how to convert the abstract information of materials’ crystal structure, chemical composition, and local atomic environment into numerical features that can be processed efficiently by computers has become the core topic of materials informatics.

**Figure 10:**
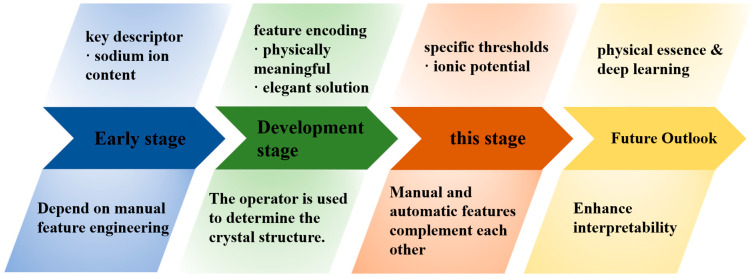
The development process and future of machine learning.

In the past, as shown in Figure 10, sodium ion content was widely used as a key descriptor. Subsequently, the introduction of physical and chemical parameters such as ionic potential and ionic electronegativity index provided a more physically meaningful and elegant solution for feature encoding, significantly improving the learning efficiency and interpretability of the model. Ionic potential [48], as a linear discriminant feature, is particularly prominent in its operator application. By setting specific thresholds, the crystal structure of the material can be directly determined. This clear physical image gives it a stronger visualization ability and interpretability than complex neural networks. Currently, ionic potential is more often used as an additional node feature, integrated with the geometric features automatically extracted by graph neural networks to achieve complementary advantages. In the near future, by numerically representing its physical essence and deeply integrating it with the attention mechanism in deep learning, ionic potential is expected to retain physical interpretability while further enhancing the ability of deep learning models to capture complex material structures, achieving more long-term development.

## 6. Synthesis Optimization: Bridging the Gap Between Theory and Experiment

The electrochemical performance of layered oxide cathode materials is not only determined by their intrinsic crystal structure and electronic structure, but is also largely influenced by the process parameters and preparation routes during synthesis [127]. Even if an ideal structure with high stability and high capacity is theoretically designed, if the synthesis conditions are not properly controlled, it may lead to the formation of inclusions, element segregation, grain coarsening or uneven distribution of sodium vacancies, which will seriously affect the actual performance of the materials. Therefore, it is necessary to deeply couple the structure design with the synthesis process and, through precise control of calcination conditions, local concentration distribution and synthesis methods, achieve the directional regulation of the material’s microstructure, thereby realizing the closed-loop optimization of “theoretical design—precise synthesis—performance realization”. This chapter systematically elaborates on three key synthesis optimization strategies guided by structure design: changing calcination conditions [128,129], regulating local concentration [130], and innovating synthesis methods [131], aiming to achieve the high-fidelity reproduction of the theoretically predicted structure at the experimental level and improve the phase purity, crystallinity, and electrochemical uniformity of the material.

### 6.1. Based on the Optimized Synthesis Method

The synthesis method is critical to realizing a material’s designed properties, particularly in ensuring compositional consistency and controlling macroscopic morphology. Each synthesis route offers distinct advantages, making it a key consideration when selecting optimal preparation conditions.

#### 6.1.1. High-Temperature Solid-Phase Method

The high-temperature solid-state method remains the most widely used and straightforward approach to synthesizing layered oxide cathodes. It typically involves mixing stoichiometric amounts of a sodium source (e.g., Na_2_CO_3_) with transition metal compounds—such as MnO_2_, NiO_2_, or doped oxides/carbonates—followed by prolonged calcination at temperatures above 800 °C. This method can be traced back to the work of M. Minakshi et al., who introduced a certain amount of alkali metal ions into MnO_2_ materials, thereby enhancing the specific capacity and cycling stability of MnO_2_ [132]. The reaction proceeds via solid-state diffusion, where atomic mobility and interfacial contact between precursors dictate the kinetics of phase formation. Several factors critically influence the outcome: the intimacy of precursor mixing, particle size distribution, and the furnace atmosphere (e.g., air vs. O_2_). Due to inherently limited diffusion in the solid state, the resulting materials often exhibit irregular, polygonal morphologies with broad particle size distributions and insufficient elemental homogeneity—particularly in multi-component systems, where local segregation of transition metals is common.

Although the products generally display high crystallinity, precise control over grain size and morphology remains challenging. More importantly, compositional inhomogeneity frequently degrades electrochemical performance, manifesting as poor cycling stability and sluggish rate capability. These non-uniform regions are also more prone to detrimental phase transitions and parasitic side reactions during electrochemical cycling. Thus, the fundamental limitation of the conventional solid-state route lies in the intrinsic trade-off between high-temperature processing and compositional uniformity.

Nevertheless, the solid-state method continues to play a vital role—not only in laboratory-scale exploration of new cathode compositions but also in industrial production. To mitigate the aforementioned issues, strategies such as multi-step calcination or pre-forming intermediate phases have been developed to enhance material consistency and structural stability.

**Figure 11:**
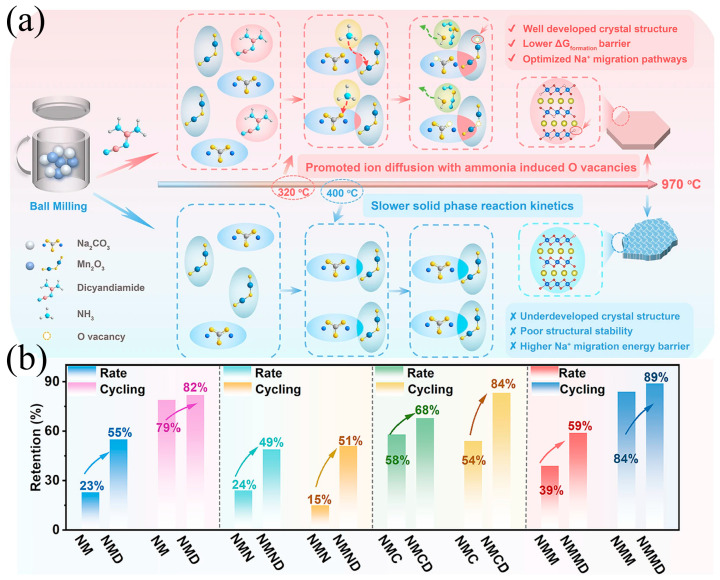
(**a**) Design principle for ammonia-induced synthesis approach of Na_1/2_MnO_2_. (**b**) The performance enhancement summary of the samples [15].

By altering the reaction atmosphere or introducing reaction additives, the effective reaction temperature can be reduced, elemental diffusion can be promoted, and crystal preferential growth can be guided [129]. Zhu et al. [15] added dicyandiamide as the ammonia source to the precursor. During the calcination process, the decomposition of dicyandiamide generates NH_3_, which forms a reducing atmosphere (Figure 11a). This not only creates a mild environment for crystal growth and inhibits excessive oxidation and volatilization of manganese, but also may form intermediate complexes with transition metals, promoting atomic migration and ordered arrangement. This strategy is highly versatile and can significantly enhance the crystallinity and structural integrity of various manganese-rich layered oxides, enabling the materials to exhibit excellent rate performance (maintaining approximately 100 mAh·g^−1^ capacity at a 5C) and cycling stability (Figure 11b). From a mechanistic perspective, this optimized solid-phase method intervenes in the thermodynamic and kinetic processes of high-temperature solid-phase reactions through atmosphere chemistry. The reducing atmosphere can stabilize the intermediate valence state of manganese, inhibit excessive formation of oxygen vacancies, and the transmission of gaseous intermediate products can provide additional quality transfer channels, allowing the components to mix more uniformly at the atomic scale, thereby obtaining products with high crystallinity and low defect concentration at relatively lower calcination temperatures.

#### 6.1.2. Sol–Gel Method

The sol–gel method is a wet chemical technique that typically uses metal alkoxides or inorganic salts as precursors. These precursors undergo hydrolysis and condensation in solution to form a homogeneous sol, which is then converted into a gel through solvent evaporation or chemical reactions. The final product is obtained after drying and calcination. The core of this process lies in achieving uniform mixing at the molecular or nanoscale level. Chelating agents such as citric acid and ethylenediaminetetraacetic acid are commonly used to ensure uniform distribution of metal ions and inhibit phase separation.

Materials produced via the sol–gel method exhibit highly uniform chemical compositions, fine particle sizes with narrow size distributions, and can be tailored to specific surface areas and pore structures by adjusting parameters such as pH, temperature, and the gelation process. The resulting products are generally of high purity and crystallize at lower temperatures compared to solid-state methods. This superior homogeneity facilitates more uniform sodium ion intercalation/deintercalation, reducing local stress and enhancing structural stability and cycling performance. Additionally, the smaller particles shorten ion diffusion paths, improving rate capability.

From a mechanistic standpoint, the sol–gel process can be regarded as a strategy for engineering a “chemically frozen” state, wherein the robust three-dimensional gel network immobilizes metal ions in close proximity, thereby suppressing elemental segregation during subsequent thermal treatment.

**Figure 12:**
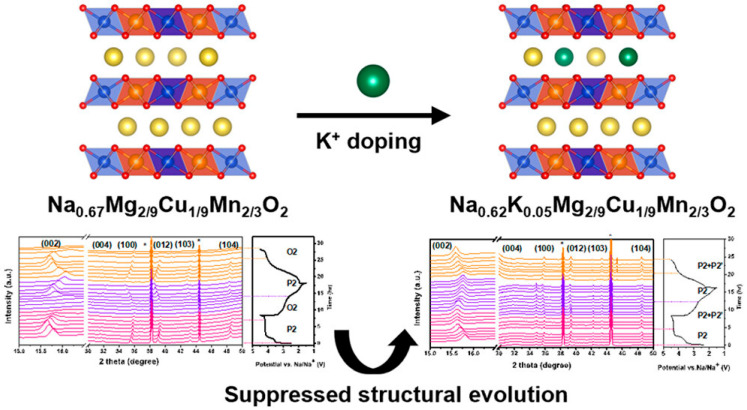
Structural stabilization of layered sodium oxide cathode via K^+^ doping [133].

Yeh et al. [133] synthesized Na_0.62_K_0.05_Mg_2/9_Mn_2/3_Cu_1/9_O_2_ via a citric acid-assisted sol–gel route, yielding a phase-pure material with exceptional structural homogeneity, as evidenced by the sharp and well-defined diffraction peaks in Figure 12. This high degree of crystallinity and phase uniformity established a robust foundation for subsequent investigations into its structural evolution and oxygen redox behavior during electrochemical cycling.

Manikandan et al. [134] synthesized Na_0.5_Ni_0.25_Mn_0.75_O_2_ via the sol–gel route. Although this composition is often considered borderline for forming a well-defined layered structure, it delivered a reversible specific capacity of 210 mAh·g^−1^ under a 0.1C rate within the voltage window of 1.5–4.0 V vs. Na^+^/Na. X-ray diffraction (XRD) patterns were collected for both the gel-derived precursor after decomposition at 400 °C for 5 h and the final product after calcination at 900 °C for 12 h. Rietveld refinement of the high-temperature product confirmed the formation of a single-phase material with nominal stoichiometry Na_0.5_Ni_0.25_Mn_0.75_O_2_, the XRD pattern of the 400 °C sample exhibits a broad hump characteristic of an amorphous phase, whereas the 900 °C sample displays sharp, intense Bragg peaks—indicative of high crystallinity and phase purity. These results underscore that the sol–gel method, when coupled with appropriate calcination conditions, enables the synthesis of highly crystalline layered oxides with excellent structural homogeneity.

#### 6.1.3. Coprecipitation Method

Coprecipitation stands as the most industrially viable synthesis route for layered oxide cathodes. In this approach, aqueous solutions of soluble transition metal salts (e.g., sulfates or nitrates) are mixed with a precipitating agent—typically NaOH or Na_2_CO_3_—under tightly controlled conditions of pH, temperature, stirring rate, and feed strategy to yield a homogeneous coprecipitated precursor. This precursor is then filtered, washed, dried, and finally calcined with a sodium source to form the target oxide.

The essence of the process lies in mastering nucleation and crystal growth kinetics in solution. By tuning supersaturation levels, adding surfactants, or modulating mixing dynamics, one can precisely engineer the morphology, particle size, and tap density of the precursor. The resulting cathode materials typically consist of spherical or near-spherical secondary particles—agglomerates of densely packed primary nanocrystals. This hierarchical architecture offers a compelling balance: the secondary spheres enable high tap density (boosting volumetric energy density), while the nanoscale primary grains shorten Na^+^ diffusion pathways, enhancing rate capability. Moreover, elemental distribution can be finely tuned at the sub-particle level, and the spherical morphology promotes uniform slurry coating and high electrode calendering density. Critically, the porous yet robust secondary structure also accommodates volume changes during cycling, mitigating mechanical degradation.

**Figure 13:**
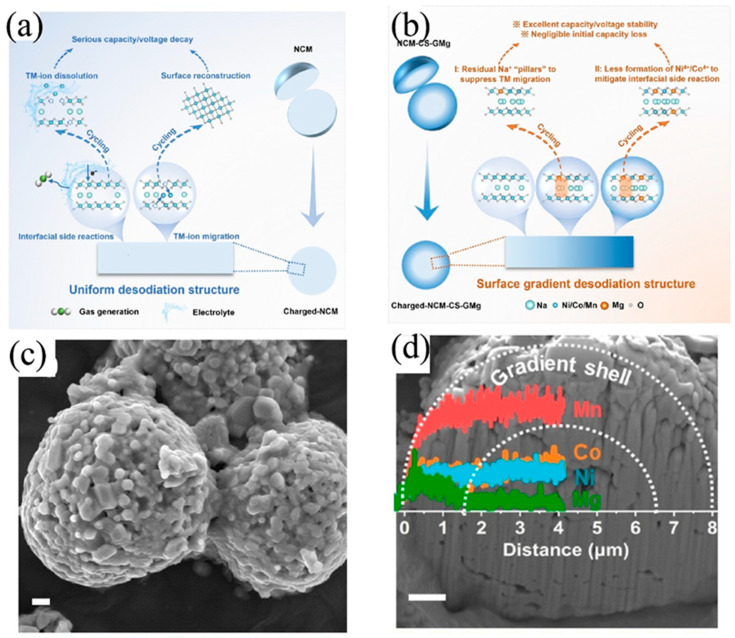
(**a**) Schematic illustration of the degradation mechanism in NCM. (**b**) Surface gradient desodiation structural design concept for NCM-CS-GMg. (**c**) SEM image. (**d**) Elementary-line scanning of the cross-section from the shell to the core regions. The scale bars in (**c**,**d**) are 1 μm [135].

The most advanced evolution of coprecipitation moves beyond the conventional paradigm of uniform precipitation. Instead, it leverages spatiotemporal programming of the precipitation process to create compositionally graded architectures within individual particles. Zhang et al. [135] introduced all metal ions simultaneously; they first precipitated a Ni/Co-rich core (Figure 13a), then gradually shifted the feed composition to enrich the outer shell with more electrochemically stable Mn (and Mg) (Figure 13b). After calcination, this yielded a core–shell cathode—Na_0.72_Ni_0.2_Co_0.21_Mn_0.55_Mg_0.036_O_2_—with a deliberately engineered compositional gradient.

Mechanistically, this design exploits differences in the hydrolysis/pH-dependent precipitation behavior of transition metal ions and their distinct diffusion kinetics. By dynamically adjusting local supersaturation and ion availability during particle growth, a continuous elemental gradient is written into the particle. This architecture delivers dual functionality (Figure 13c,d): The Ni/Co-rich core provides high specific capacity, while the Mn-rich, Na-deficient outer shell enhances interfacial stability. The Mn-rich surface suppresses irreversible phase transitions and parasitic reactions with the electrolyte. More subtly, the Na-deficient shell acts as an intrinsic sodium buffer zone, alleviating structural stress during deep desodiation in early cycles by moderating the concentration gradient of extracted Na^+^ ions across the particle.

In essence, this approach transcends incremental optimization—it represents a materials-by-design philosophy, where performance and stability are co-engineered from the atomic to the particulate scale, offering a powerful blueprint for next-generation sodium-ion cathodes.

#### 6.1.4. Comparison Between Sol–Gel Method and Co-Precipitation Method

The sol–gel method and the co-precipitation method are the two most commonly used wet chemical synthesis strategies for preparing layered oxide cathode materials for sodium-ion batteries, each suited for different research and development objectives. The sol–gel method achieves highly uniform mixing of precursors at the molecular/atomic scale, making it particularly suitable for systems with complex compositions and stringent requirements for elemental distribution uniformity (e.g., high-entropy oxides). The resulting products typically exhibit nanoscale particle sizes and high specific surface areas, facilitating thorough solid-state reactions and precise control of defect structures. However, this method involves cumbersome process steps, higher raw material costs, and is prone to cracking during gel drying in large-scale production, limiting its industrial application.

In contrast, the co-precipitation method, with its excellent scalability and morphological controllability, can directly synthesize precursor microspheres with high sphericity and tap density, significantly improving the volumetric energy density and processing performance of electrodes. It has become the mainstream route for industrialization. The key challenge lies in precisely matching the precipitation kinetics of multiple metal ions during co-precipitation to avoid local compositional inhomogeneity and phase separation caused by elemental segregation.

Therefore, the sol–gel method holds an advantage in fundamental research when exploring novel complex compositions or pursuing atomic-level uniformity. On the other hand, for practical cathode material development, the co-precipitation method offers a better balance between performance, cost, and process stability.

### 6.2. Synthesis Optimization Based on Calcination Conditions

Calcination is the core step in the solid-phase synthesis of layered oxides, and its temperature, time, heating rate and atmosphere directly determine the phase structure, grain size, defect concentration and sodium content of the final product. By combining thermogravimetric-differential scanning calorimetry (TG-DSC) analysis and in situ high-temperature XRD characterization, a temperature-phase transformation mapping relationship can be established, and then the calcination regime can be optimized to match the formation kinetics of the target structure.

Therefore, the current calcination method and the structure of the precursor materials have become an important way to achieve the theoretical design method. At the same time, the environment of calcination and the cooling method also play a crucial role. Unlike the principle of designing different phase structures through ion diffusion, as mentioned earlier, the environment and cooling method of calcination form a unique phase structure by constructing the environmental differences on the surface of the material and the interior of the phase body. Elisa Grépin et al. [136] explored how variations in the calcination atmosphere (CO_2_, Ar, Air, Synthetic Air, O_2_) and cooling method affect the phase structure of Na_0.85_Ni_0.38_Zn_0.04_Mn_0.48_Ti_0.1_O_2_. Under slow cooling in air, a mixed O3 + P2 structure formed at 900 °C (6 h, Na:Mn = 0.85), while raising the temperature to 1000 °C under the same atmosphere yielded a pure O3 phase. Notably, rapid quenching resulted in a mixed Mn^3+^/Mn^4+^ valence state, whereas slow cooling preserved a single oxidation state. Reheating to 1000 °C (1 h) followed by quenching-induced transition metal and oxygen vacancies in Ar, Air, Synthetic Air, and O_2_ atmospheres, while slow cooling produced multiphase structures. XRD analysis revealed the distribution of O-type and P-type phases, with the O3 and P3 structures sharing a proportion of the same (003) crystal planes. The average homogeneity was 40.77% for O3 and 37.95% for P2, indicating that P3 and O3 coexist as symbiotic phases. Electrochemical analysis further showed that rapid quenching significantly lowered the open-circuit voltage, likely due to introduced transition metal and oxygen vacancies, which reduced the water-adsorption energy. Quenching also led to irreversible capacity loss, attributed to irreversibility near 4.3 V, and decreased stable capacity as a result of multiphase behavior. Thus, rapid quenching proved ineffective for synthesis in this system. Although the electrochemical performance did not reach ideal levels, the approach offers innovative insights into material synthesis, underscoring the critical role of calcination temperature in governing crystal structure.

**Figure 14:**
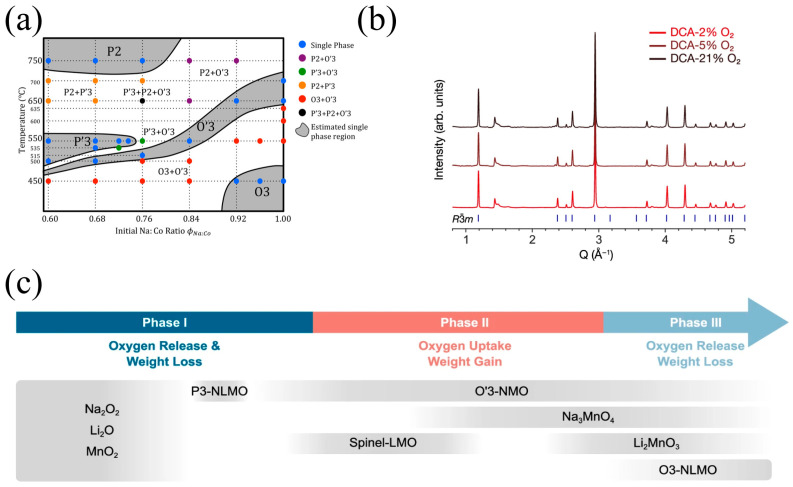
(**a**) Synthesis phase diagram of Na_x_CoO_2_ as a function of the precursor Na:Co ratio $\Phi_{Na:Co}$ (*X* axis) and the sintering temperature (*Y* axis) [137]. (**b**) XRD results of O3-NLMO synthesized via dynamically controlled atmosphere containing 2%, 5% and 21% O_2_ for the ramping process, respectively. (**c**) A summary of the reaction processes and intermediate compounds during the temperature ramping [138].

Lei et al. [137] provided compelling experimental evidence for the thermodynamic dependence of phase selection on sodium content and synthesis temperature in layered Na–Co oxides. As illustrated in Figure 14a, elevated temperatures combined with lower Na:Co ratios favor the stabilization of the P-type phase, whereas lower temperatures and higher sodium concentrations promote the formation of the O-type phase. Notably, the O3′ phase exhibits a broad compositional tolerance, remaining accessible even under relatively sodium-deficient conditions—a feature that underscores its structural robustness across a wide stoichiometric window.

In order to investigate the influence of the calcination atmosphere on the phase formation process of the material, Qiu et al. [138] employed in situ X-ray diffraction (XRD) to elucidate the critical role of calcination atmosphere in the synthesis of O3-Na[Li_1/3_Mn_2/3_]O_2_. By dynamically modulating the oxygen partial pressure during thermal treatment, they directly correlated atmospheric conditions with phase evolution and impurity formation. As shown in Figure 14b, an oxygen-rich environment promotes the emergence of undesired secondary phases, likely due to over-oxidation of Mn^3+^ or kinetic trapping of metastable intermediates.

Through temperature-resolved in situ XRD (Figure 14c), the authors delineated a three-stage crystallization pathway. Stage I (RT → 290 °C): Initial nucleation of the layered framework, accompanied by significant oxygen release from precursor decomposition; Stage II (290 → 550 °C): critical incorporation of Li^+^ into the transition metal layer, concurrent with oxygen uptake—a signature of redox-driven structural ordering; and Stage III (550 → 700 °C): final phase maturation, marked by a second oxygen evolution event and the establishment of long-range crystalline order in the O3 structure. This mechanistic insight highlights how precise control over both the atmosphere and thermal profile is essential to suppress parasitic phases and achieve phase-pure, high-performance cathodes.

Song et al. [139] employed a co-precipitation method to prepare precursors, which were then calcined at varying temperatures (e.g., 800 °C, 900 °C, 1000 °C) and durations (12 h, 15 h, 18 h) to synthesize a series of samples with an optimized concentration-gradient distribution. Their results indicate that extended calcination promotes a more homogeneous elemental distribution within the material and markedly enlarges the interlayer spacing (d-spacing) and unit-cell volume of the sodium layer. This expansion creates wider channels for Na^+^ migration, thereby reducing the diffusion energy barrier.

### 6.3. Synthetic Optimization Method Based on Local Concentration

As noted previously, the structural characteristics of a material are influenced not only by its intrinsic design (discussed in Section 4), but also considerably by the synthesis method employed. This is particularly evident in terms of synthetic kinetics: rapid high-temperature kinetics enable high compositional uniformity to be achieved at relatively low temperatures and within short timeframes. In contrast, ions with sluggish high-temperature kinetics—especially those with high charge and large ionic radius—possess higher ionic potential and stronger interactions. As a result, achieving a homogeneous distribution becomes more challenging, often leading to local concentration variations. Consequently, such systems tend to favor the formation of coexisting P- and O-type phases.

The aforementioned perspective was proposed by Ma et al. [140], who investigated two precursors with irregular and regular morphologies under identical calcination conditions to synthesize O3-NaNi_0.4_Fe_0.2_Mn_0.4_O_2_ cathode materials. They observed that irregular precursors undergo non-uniform sodium-ion diffusion. During solid-state sodiation, a shell with an $R_{3}^{-}m$ structure forms, encapsulating a large rock-salt phase core. This leads to undesirable severe phase transitions and void formation in subsequent calcination steps. In contrast, spherical or uniformly shaped precursors can mitigate the influence of sodium-ion diffusion pathways on phase formation, thereby better preserving the advantages of the designed structure.

**Figure 15:**
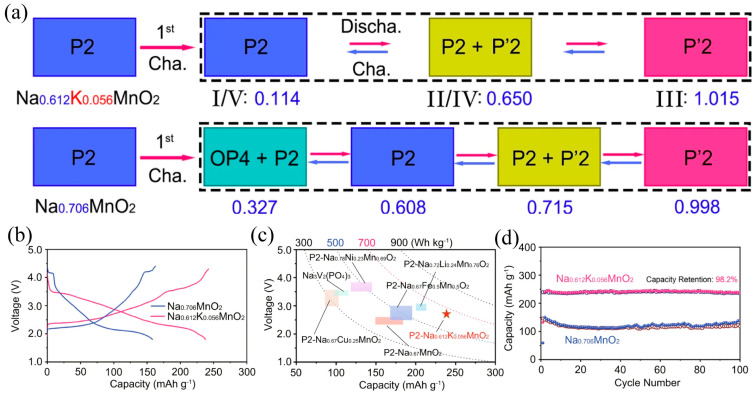
(**a**) Schematic illustration of phase transitions along with the content change in Na^+^ in Na_0.612_K_0.056_MnO_2_ and Na_0706_MnO_2_ in cycles of charge and discharge. (**b**) Typical charge/discharge curves of Na_0.612_K_0.056_MnO_2_ and Na_0.706_MnO_2_ at 20 mA g^−1^ in the third cycle. (**c**) Comparison of energy density and average voltages of Na_0.612_K_0.056_MnO_2_ with typical cathode material. (**d**) Cycle performance of Na_0.612_K_0.056_MnO_2_ and Na_0.706_MnO_2_ at 50 mA g^−1^ [142].

These findings indicate that conventional sodium-ion battery calcination—which typically introduces sodium into precursors via high-temperature treatment—often yields phase structures with non-uniform sodium content. This variability stems from differences in sodium-ion diffusion pathways or limited diffusion kinetics during synthesis. Notably, such non-uniformity can be leveraged as a novel mechanism for designing biphasic structures. In a related approach, Wang et al. [141] synthesized O3-NaNi_0.5_Mn_0.5_O_2_ (O3-NNMO) and then deposited a sodium-rich P2-type Na_0.8_Ni_0.33_Mn_0.67_O_2_ layer onto its surface via a sol–gel method, obtaining an O3@P2 core–shell composite. The hybrid phase enables simultaneous high capacity and improved cycling stability, as reflected in the charge–discharge profiles. Through relatively low-temperature calcination, a dual-phase structure with differing sodium concentrations in the surface and bulk can be achieved, further optimizing sodium storage behavior. Wang et al. [115] introduced electrochemically inactive alkali metal ions (e.g., K^+^) into the prismatic Na sites of P2-type layered oxides to deliberately create Na vacancies, thereby tailoring the local chemical environment within the transition metal and alkali metal layers. Through this strategy, they successfully synthesized a high-performance cathode material with the composition Na_0.612_K_0.056_MnO_2_. As illustrated in Figure 15a, the material exhibits both high specific capacity and excellent cycling stability, which are primarily attributed to the suppression of detrimental phase transitions during (de)intercalation—particularly the absence of the OP4 phase commonly observed in pristine Na_0.706_MnO_2_. This structural distinction is further corroborated by the voltage profiles in Figure 15b, whereas Na_0.706_MnO_2_ displays pronounced charge–discharge plateaus indicative of multi-step phase transformations, Na_0.612_K_0.056_MnO_2_ exhibits a more sloping profile with significantly reduced plateau regions, reflecting a solid-solution-like reaction mechanism. Consequently, the minimized involvement of first-order phase transitions not only enhances structural reversibility but also contributes to a higher practical specific energy and improved cycle life, as demonstrated in Figure 15c,d.

**Figure 16:**
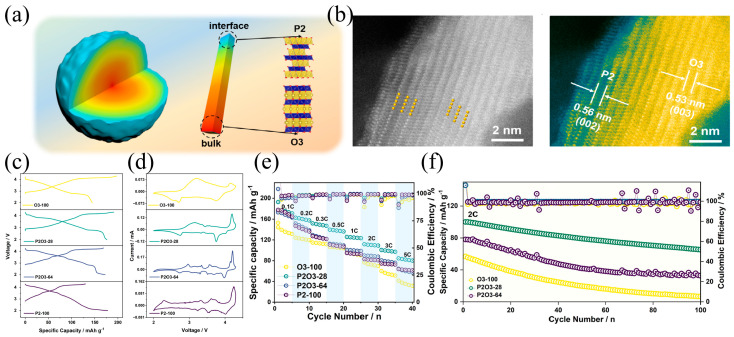
(**a**) Schematic diagram of P2O3 heterostructure layered oxide. (**b**) HAADF-STEM image and corresponding colored pattern of P2O3-28. (**c**) Galvanostatic charge/discharge curves for the first cycle at 0.1C in 2.0–4.3 V. (**d**) CV curves at 0.1 mV·s^−1^. (**e**) Rate performance in 2.0–4.3 V. (**f**) Cycling performance of O3-100 (yellow), P2O3-28 (blue) and P2O3-64 (purple)during 100 cycles at 2C [143].

In contrast, Kong et al. [143] formed the phase through the external precipitation of Na:Mn = 0.44:1 and finally calcined it at 800 °C for 15 h. Their research found that the external structure was different from the rod-like structure formed by the separate calcination of Na:Mn = 0.44:1 material, mainly forming the P2 phase structure (Figure 16a,b). This method avoids the form of lattice misalignment that may be caused by different phases, achieving a synergistic effect that is conducive to the stability of the structure and showing high energy density (based on the cathode active material as 587.34 Wh·kg^−1^). As shown in Figure 16c–f, the cyclic voltammetry (CV) curves reveal that the P2/O3 biphasic material exhibits significantly smoother redox behavior compared to the single-phase O3 counterpart. Specifically, within the voltage window of 2.5–3.5 V, the redox peaks of the biphasic system—particularly for the P2O3-28 sample—are broader, less intense, and more continuous, indicating the suppression of sharp first-order phase transitions. This suggests that the coexisting P2 and O3 phases act as mutual structural buffers during (de)intercalation, effectively mitigating abrupt volume changes and voltage hysteresis typically associated with single-phase systems. Consequently, the material demonstrates enhanced electrochemical reversibility, improved rate capability, and more stable cycling performance, as evidenced by the galvanostatic charge–discharge profiles in Figure 16e,f.

Among the doped compositions, the sample with 28% P2-phase content (P2O3-28) delivers the best overall performance, exhibiting superior structural integrity, excellent air stability, and outstanding full-cell electrochemical metrics. Further microscale stress simulations confirm that the P2-rich surface layer effectively redistributes and alleviates mechanical stress imposed on the interior O3 domains during cycling. This protective effect suppresses plastic deformation and delays structural degradation, thereby significantly enhancing the long-term mechanical and electrochemical stability of the cathode.

## 7. Summary and Outlook

This article systematically presents the progress in the rational design of manganese-based layered oxide cathode materials for sodium-ion batteries. From a thermodynamic perspective, it reveals the regulatory mechanism of sodium content and transition metal ion potential on the P2/O3 phase structure. At the elemental chemistry level, it analyzes the influence of the electronic structure, oxidation state, and bonding characteristics of transition metals on the electrochemical performance of the materials, and clarifies the synergistic mechanism between active and inert metals. The high-entropy strategy effectively suppresses Jahn–Teller distortion and harmful phase transitions through configurational entropy increase, lattice retardation, and “cocktail effect”, thereby enhancing the material stability. Furthermore, by combining machine learning and multi-dimensional descriptors, high-throughput prediction and screening of phase behavior in the complex component space are achieved, promoting data-driven material design. Finally, by optimizing the calcination process, constructing concentration gradients, and forming P2/O3 heterostructures, the theoretical structure is accurately transformed with high fidelity, significantly improving the cycling stability and comprehensive electrochemical performance of the materials.

Nevertheless, several critical challenges remain unresolved. Conventional descriptors (e.g., ionic potential) exhibit limited physical applicability in high-entropy, compositionally complex systems, struggling to accurately capture local chemical environments and dynamic electronic structure evolution. A persistent gap exists between theoretical predictions and experimental synthesis, particularly regarding atomic-scale dopant feasibility and phase-purity control. Moreover, a unified criterion linking structure to performance in high-entropy materials is still lacking, and the mechanisms for multi-objective performance co-optimization remain poorly understood.

Moving forward, cathode material design must prioritize the development of atomically localized dynamic descriptors that are both physically interpretable and adaptive to electrochemical environments. Integrating graph neural networks with attention mechanisms offers a promising approach to decode complex local coordination effects. Moreover, beyond intrinsic material stability, the actual electrochemical conditions and material interfaces play decisive roles. Therefore, surface/interface engineering represents a rational and effective strategy for enhancement. Concurrently, a closed-loop integration of theory, synthesis, and characterization—guided by in situ techniques and multiscale simulations—is essential to enable precise synthesis. This holistic approach is critical for achieving practical breakthroughs in developing stable, high-energy-density, and low-cost sodium-ion cathode materials.

## Figures and Tables

**Table 1:** The relationship between material structure and sodium content.

Materials	Sodium Content	Phase	Reference
Na_2/3_Ni_1/3_Mn_2/3_O_2_	0.67	P2	[24]
Na_0.696_Ni_0.329_Mn_0.671_O_2_	0.696	P2	[24]
Na_0.67_Mn_0.45_Ni_0.22_Co_0.33_O_2_	0.67	P2	[25]
Na_0.67_Mn_0.55_Ni_0.21_Co_0.24_O_2_	0.67	P2	[26]
Na_0.67_Mn_0.45_Ni_0.18_Co_0.24_Ti_0.1_Mg_0.03_O_2_	0.67	P2	[26]
Na_0.67_Mn_0.45_Ni_0.18_Co_0.18_Ti_0.1_Mg_0.03_Al_0.04_Fe_0.02_O_2_	0.67	P2	[26]
Na_0.67_Mn_0.8_Fe_0.1_Ni_0.1_O_2_	0.67	P2	[27]
Na_0.67_Mn_0.8_Fe_0.1_Mg_0.1_O_2_	0.67	P2	[27]
Na_2/3_Mn_1/2_Ni_1/6_Co_1/3_O_2_	0.67	P2	[14]
Na_0.7_Ni_0.2_Mn_0.6_Cu_0.15_Ti_0.05_O_2_	0.7	P2	[28]
Na_0.67_Li_0.1_Mn_0.62_Fe_0.18_Cu_0.09_Sm_0.01_O_2_	0.67	P2	[29]
Na_0.67_Zn_0.05_Ni_0.15_Fe_0.20_Mn_0.60_O_1.95_F_0.05_	0.67	P2	[30]
Na_0.67_Ni_0.15_Fe_0.20_Mn_0.65_O_2_	0.67	P2	[30]
Na_0.85_Li_0.08_Mg_0.04_Ni_0.22_Al(B)_0.04_Mn_0.62_O_2_	0.85	P2	[31]
Na_0.70_Ni_0.20_Cu_0.15_Mn_0.575_Ti_0.075_O_2_	0.7	P2	[32]
Na_0.70_Ni_0.20_Cu_0.15_Mn_(0.65−x)_Ti_x_O_2_	0.7	P2	[32]
Na_0.70_Ni_0.20_Cu_0.15_Mn_0.65_O_2_	0.7	P2	[32]
NaMn_4.58_Al_18_Ni_2.58_O_2_	1	P2	[33]
NaNi_1/3_Fe_1/3_Mn_1/3_O_2_	1	O3	[34]
Na_x_Li_0.05_Ni_0.45_Mn_0.25_Mg_0.05_Ti_0.25_O_2_	0.7–0.95	O3	[35]
Na_0.85_Mn_0.45_Ni_0.25_Li_0.05_Cu_0.1_Ti_0.15_O_2_	1	O3	[36]
Na_0.98_Ca_0.01_Ni_0.33_Fe_0.33_Mn_0.315_Sn_0.015_O_2_	0.98	O3	[37]
NaNi_0.475_Mn_0.475_Mo_0.05_O_2_	1	O3	[38]
Na(Ni_0.4_Cu_0.1_Mn_0.4_Ti_0.1_)_0.92_Fe_0.08_O_2_	1	O3	[39]
Na_0.9_Mn_1/2_Fe_1/3_Cu_1/6_O_2_	0.9	O3	[40]
NaNi_0.35_Fe_0.2_Mn_0.3_Ti_0.1_Sb_0.05_O_2_	1	O3	[41]
NaNi_0.305_Fe_0.33_Mn_0.33_Ce_0.025_O_2_	1	O3	[41]
Na_0.95_Li_0.07_Sn_0.01_Ni_0.22_Fe_0.2_Mn_0.5_O_2_	0.95	O3	[42]
NaLi_1/3_Mn_2/3_O_2_	1	O3	[43]
NaNi_0.3_Mn_0.52_Mo_0.03_Cu_0.1_Ti_0.05_O_2_	1	P2/O3	[44]
NaLi_0.05_Fe_0.04_Al_0.01_Ni_0.4_Mn_0.4_Ti_0.1_O_2_	1	O3	[45]
Na_x_Li_0.05_Mn_0.55_Ni_0.3_Cu_0.075_Mg_0.025_O_2_	0.7–1	P2/O3	[46]

**Table 2:** Common descriptors.

Descriptor Symbol	Implication	Definition and Computational Logic
$n_{Na}$	Sodium Concentration	The content of sodium ions in the material phase
$\Phi_{TM}$	TM Ionic Potential	The electrostatic interaction strength between the transition metal (TM) cation and oxygen anion. It is calculated as the ratio of the effective charge of the TM ion ($Z_{TM}$) to its ionic radius ($r_{TM}$): $\Phi_{TM}=Z_{TM}/r_{TM}$.
$S_{TM}$	TM Mixing Entropy	The configurational entropy of the transition metal layer. It quantifies the disorder introduced by multiple TM elements and is calculated using the Boltzmann equation, $S_{TM}=-R\sum_{i=1}^{n}c_{i}lnc_{i}$, where $c_{i}$ is the molar fraction of the *i*th element and *R* is the gas constant.
$\Phi_{cation}$	Cationic Potential	A composite descriptor representing the overall electrostatic field of all cations. It is typically defined as the weighted average of the ionic potentials of all cations in the lattice, used to predict structural stability and phase formation.
${Ι}_{TM}$	TM Ionization Energy	The energy required to remove an electron from the transition metal ion (oxidation potential). It reflects redox activity and is usually defined as the first ionization energy of the isolated gaseous atom, often used as a proxy for Fermi level alignment.
$r_{TM}$	TM Radius	The ionic radius of the transition metal cation. It is a critical geometric parameter that influences the TM–O bond length and the lattice volume; values are typically taken from Shannon’s empirical tables for specific coordination numbers (e.g., octahedral sites).

**Table 3:** Method for synthesizing P2/O3 mixed phase.

Materials	Method	Specific Method	Reference
Na_1−x_Li_x_Ni_0.5_Mn_0.5_O_2_	Doping	Li doped alkali metal layer	[95]
Na_0.67_Mn_0.55_Ni_0.25_Ti_0.2−x_Li_x_O_2_	Doping	Li-doped transitional metal layer	[97]
Na_x_[Ni_0.6_Co_0.2_Mn_0.2_]O_2_	Synthetic method	Using co-precipitation with hydroxides	[98]
P2-Na_0.7_MnO_2_/O3-NaMnO_2_	Synthetic method	Change the crystallization cooling rate	[99]
Na_0.67_Ni_0.33_Mn_0.57_Sn_0.1_O_2_	Doping	Doping with Sn element	[100]
Na_0.8_Li_0.2_Fe_0.2_Mn_0.6_O_2_	Doping	Li doping	[101]
Na_0.7_Ni_0.2_Cu_0.1_Fe_0.2_Mn_0.5_O_2–δ_	Doping	Introducing impurities to create local differences in cation potential	[102]
Na_0.732_Ni_0.273_Mg_0.096_Mn_0.63_O_2_	Doping	Mg ion doping	[103]
Na_0.7_Mn_0.4_Ni_0.3_Cu_0.1_Fe_0.1_Ti_0.1_O_1.95_F_0.1_	Synthetic method	Change the calcination temperature	[104]
Na_x_Li_0.05_Mn_0.55_Ni_0.3_Cu_0.075_Mg_0.025_O_2_	Doping	-	[46]
Na_0.85_Li_0.05_Ni_0.3_Fe_0.1_Mn_0.5_Ti_0.05_O_2_	-	-	[105]
Na_x_Cu_0.1_Co_0.1_Ni_0.25_Mn_0.4_Ti_0.15_O_2_	-	Change the sodium ion content	[106]
Na_0.76_Ni_0.20_Mn_0.42_Fe_0.30_Mg_0.04_Ti_0.015_Zr_0.025_O_2_	Doping	Mg, Ti, Zr doping	[107]
Na_0.75_Cu_0.1_Fe_0.2_Mg_0.2_Mn_0.4_Ti_0.1_O_2_	Doping	Forming high-entropy materials by incorporating multiple elements	[108]
Na_0.8_Ni_0.23_Fe_0.34_Mn_0.43_O_2_	Doping	Fe, Ni doping	[109]
Na_0.8_Li_0.03_Mg_0.05_Ni_0.28_Fe_0.05_Mn_0.54_Ti_0.05_O_2_	Doping	Forming high-entropy materials by incorporating multiple elements	[110]
Na_x_Mn_0.4_Ni_0.3_Fe_0.15_Li_0.1_Ti_0.05_O_2_	Doping	Forming high-entropy materials by incorporating multiple elements	[23]
Na_0.8_Mg_0.06_Ni_0.34_Mn_0.54_Ti_0.06_O_2_	-	-	[111]
Na_0.76_Ni_0.33_Mn_0.48_O_2_	Synthetic method	Coprecipitation	[112]

## Data Availability

No new data were created or analyzed in this study. Data sharing is not applicable to this article.
